# Novel Antithrombotics in Geriatric PCI for STEMI: Balancing Ischemic Protection with Bleeding Risk: Short title: Novel Antithrombotics in Geriatric PCI for STEMI

**DOI:** 10.31661/gmj.v15i.4032

**Published:** 2026-06-07

**Authors:** Ibrahim Pakizeh, Bahram Sohrabi1

**Affiliations:** ^1^ Cardiovascular Research Center, Tabriz University of Medical Sciences, Tabriz, Iran

**Keywords:** Older Adults, Geriatric, Percutaneous Coronary Intervention, ST-Segment elevation Myocardial Infarction

## Dear Editor,

Older adults undergoing percutaneous coronary intervention (PCI) demonstrate disproportionately higher mortality despite evolving procedural strategies and technological advances [1]. PCI in elderly patients might be associated with increased mortality despite lower repeat revascularization risk [2], while contemporary evidence indicates that newer-generation stents, transradial access, and optimized adjunctive pharmacotherapy may improve outcomes through more individualized revascularization strategies [3]. But generally, primary PCI is preferred reperfusion for ST-Segment elevation myocardial infarction (STEMI) regardless of age, if appropriate and feasible, based on the European Society of Cardiology [4]. In some circumstances, timely PCI might not be feasible where selected elderly/frail STEMI cohorts, conservative pharmacological management without PCI, is shown to be associated with adverse outcomes. The CAMI registry (2016) identified non-PCI elderly patients to have the highest-risk of mortality, heart failure, mechanical complications, and cardiac arrest versus reperfused peers while receiving anticoagulation, nitrates, but suboptimal guideline-directed antiplatelet, statin, β-blocker, ACEi/ARB therapy [5]. An octogenarian cohort (2019) showed that conservatively managed patients were frailer, on dual antiplatelet agents, statins, diuretics, fewer β-blockers/ACEi/ARBs, but they experienced greater in-hospital mortality and poorer prognosis [6]. A 2020 cohort (979 elderly) reported non-primary PCI patients had higher in-hospital death, reinfarction, pulmonary edema, cardiogenic shock, and received more diuretics, less dual antiplatelet and ACEi, β-blockers [7]. A Spanish cohort (2003-2012) of non-reperfused patients (neither PCI nor thrombolysis, n=139,130) had 17.3% mortality vs 4.8% with PCI, relying solely on pharmacological support [8]. Pharmacoinvasive (PI) is the other alternative that represents a bleeding-sparing alternative when timely pPCI is infeasible. Emerging evidence suggests PI reperfusion may rival primary PCI (pPCI) in elderly STEMI, though not supplant it, with registry data indicating superior ST-resolution and reduced composite outcomes with tenecteplase-based PI despite longer ischemic delay [9]. Half-dose tenecteplase in elderly yields noninferior efficacy and eliminates excess intracranial hemorrhage, while dual antiplatelet regimens and anticoagulation remain integral [10]. Radial access consistently improves outcomes across PI and pPCI strategies, yet comparative superiority of PI over pPCI remains unproven and limited by clopidogrel-only protocols [11]. Collectively, P-PCI yet remains the core treatment of elderly patients with STEMI. With emergence of modern anti-thrombotic agents like P2Y12 inhibitors, these medications have also been implied in peri-PCI treatments of older adult populations. 

In elderly patients with STEMI managed with PCI or pharmacoinvasive/non-PCI strategies, modern P2Y12 inhibitors such as ticagrelor and prasugrel provide potent platelet inhibition that is hypothesized to improve coronary microcirculation and myocardial salvage while requiring careful selection due to bleeding risk, with prasugrel demonstrating superior microvascular perfusion and left ventricular recovery compared with ticagrelor in PCI-treated cohorts [12]. More potent P2Y12 inhibition, particularly ticagrelor, is associated with reduced ischemic events and a potential survival advantage compared with clopidogrel, although evidence is influenced by real-world selection bias and heterogeneous risk profiles that include frailty and comorbid burden, which may modify net clinical benefit [13]. However, this therapeutic intensification consistently increases bleeding risk, especially after hospital discharge, and therefore demands individualized risk stratification and possible dose de-escalation strategies to balance haemorrhagic complications against antithrombotic efficacy in this vulnerable population [13]. Network meta-analysis of randomized trials in adults aged ≥70 years with acute coronary syndromes suggests prasugrel provides the highest probabilistic reduction of ischaemic outcomes, while clopidogrel minimizes major bleeding and ticagrelor shows inconsistent benefit with superior stent thrombosis ranking confidence intervals definitions [14]. A systematic review and meta-analysis in general population STEMI patients (all ages) compared short-duration dual antiplatelet therapy with aspirin plus potent P2Y12 inhibitors such as ticagrelor or prasugrel followed by monotherapy versus prolonged dual therapy and demonstrated that bleeding was reduced particularly in elderly populations, while maintaining comparable ischemic outcomes [15]. Another systematic review and meta-analysis that only focused on elderly patients (aged ≥65 years) found a contrasting result that potent P2Y12 inhibitors reduced cardiovascular mortality yet increased major bleeding without a significant difference in major adverse cardiovascular events overall [16]. P2Y₁₂ inhibitors are also assessed in pre-PCI era. A retrospective cohort study evaluated P2Y₁₂ inhibitor pretreatment, including clopidogrel, ticagrelor and prasugrel, in patients undergoing PCI for STEMI that demonstrated increased risks of mortality, stent thrombosis and restenosis over one year, particularly relevant for elderly populations with higher thrombotic vulnerability and comorbidity burden, while bleeding outcomes remained unchanged [17].

In the PRAISE registry, ticagrelor demonstrated a neutral effect on all-cause mortality while modestly reducing myocardial infarction and MACE, yet this ischemic advantage was consistently counterbalanced by a significantly increased risk of major bleeding, thereby suggesting that any net clinical benefit remains fragile and highly dependent on individual haemorrhagic susceptibility rather than a uniform superiority over clopidogrel in routine elderly practice [18]. In a propensity-matched cohort of patients aged ≥75 years treated with PCI, ticagrelor was associated with a statistically significant increase in both any bleeding and clinically relevant BARC-defined haemorrhage without any meaningful reduction in MACCE, indicating a lack of efficacy gain despite intensified platelet inhibition and thereby questioning its routine use in older patients where bleeding risk predominates over ischemic modulation [19]. A systematic review and meta-analysis encompassing over 29,000 elderly ACS patients found that prasugrel or ticagrelor-based dual antiplatelet therapy increased bleeding events compared with clopidogrel without improving primary efficacy endpoints, which critically undermines the assumption of class-wide superiority of potent P2Y12 inhibition in ageing populations and highlights the persistent absence of net ischemic benefit despite pharmacological intensification [20]. Another meta-analysis focusing specifically on elderly ACS patients similarly reported that ticagrelor reduced all-cause and cardiovascular mortality but simultaneously increased major bleeding risk without significant differences in composite ischemic endpoints, thereby revealing an inconsistent and methodologically heterogeneous evidence base in which apparent survival benefits may be offset or diluted by clinically consequential haemorrhagic complications [21].

Vorapaxar, another modern anti-platelet agent, is a PAR-1 antagonist that reduces thrombin-mediated platelet activation and is approved with aspirin and clopidogrel in stable atherosclerotic disease rather than STEMI PCI. However, in elderly patients undergoing PCI for STEMI, evidence remains limited, and bleeding risk is significantly increased, warranting cautious selection and an individualized antithrombotic strategy approach [22].

Despite accumulating data, the prevailing cardiology paradigm remains intellectually stagnant and clinically reckless. Chronological age continues to serve as a flawed proxy for biological vulnerability, while aggressive antiplatelet protocols, lauded for ischemic reduction, systematically sacrifice fragile patients on the altar of statistical significance. Uncritical adoption of potent P2Y₁₂ inhibitors exposes aging bodies to inexorable hemorrhagic risk without durable survival benefits.

## Conflict of Interest

The authors affirm that there are no competing interests or conflicts of interest to report.

## AI Disclosure Statement

During the preparation of this manuscript, the authors used ChatGPT, OpenAI company for language editing, grammar improvement, and liboberry.com for reference management. After its use, the authors thoroughly reviewed, verified, and revised all AI-assisted content to ensure accuracy and originality. The authors take full responsibility for the integrity and final content of the published article.
